# Technology obsolescence across the adult lifespan in a USA internet sample

**DOI:** 10.3389/fpubh.2022.1005822

**Published:** 2022-10-06

**Authors:** Nicholas Gray, Neil Charness

**Affiliations:** ^1^Department of Psychology, Florida State University, Tallahassee, FL, United States; ^2^Institute for Successful Longevity, Florida State University, Tallahassee, FL, United States

**Keywords:** smartphone, telehealth, mobile device, computer, tablet, smartwatch, home digital assistant

## Abstract

We know that older adults are less likely to own certain technological devices, such as smartphones, a technology now integral to telehealth. However, for those older adults who do own devices, we know very little about how their devices may differ from those of younger adults. The age of a device can determine the types of programs it can run, as well as the level of protection it has against malicious code. The following study is an attempt to understand the ages of devices owned by different demographic groups. An electronic survey was sent to American adults from ages 19–97, querying the types of devices they own, how old those devices are, when they plan on replacing them, and demographic information. Regression models were employed to determine the factors that predict device ownership and the age of the devices owned. We replicate the finding that older adults are less likely to own certain devices, like smartphones and laptops. However, they may be more likely to own more dated devices, such as non-smart mobile phones. Models of device age showed that older adults are more likely to own older smartphones, as well as older desktop and laptop computers. Thus, older adults may be more susceptible to hacking, due to obsolete technology. In some cases, they also may not have devices modern enough for technology-based health interventions. Thus, obsolete devices may present an additional barrier for adoption of technology-based interventions by older adults.

## Introduction

Making the conveniences of information and communication technology (ICT) accessible to the widest range of individuals possible is a goal shared by many researchers and technology designers (1). Beyond convenience, ICT adoption also has the potential to promote healthy behavior through monitoring of health practices and implementation of technology-based interventions. Periodic surveys of technology ownership have provided estimates of the proportion of individuals in different demographic groups that have adopted certain ICTs [see (2) for USA data]. Population trends show a consistent increase in the proportion of individuals who own smartphones and use the internet year after year, even among older adults. However, for certain devices, such as smartphones, rates of ownership for older adults still lag significantly behind those of younger adults. For example, in 2021 in the USA, roughly 95% of those under the age of 50 owned a smartphone, while 61% of those aged 65 and older owned one. Yet different rates of adoption are seen for different classes of ICT devices. In 2021, 44% of those aged 65 and older owned a tablet, which is commensurate with the 46% ownership seen in those aged 18–29 (3).

Empirically tested models of technology adoption such as (4)'s Universal Theory of Adoption and Use of Technology (UTAUT), and Chen and Lou's (5) Senior Technology Adoption Model (STAM), and others [e.g., (6, 7)] have identified a large set of factors that influence adoption and use, though these may vary across technology types [e.g., (8)]. Factors can be roughly classified into costs (e.g., financial, ease of use, privacy) and benefits (e.g., perceived usefulness). Given normative changes in cognition with age [e.g., (9)] which reduce the learning rate for acquiring skill with new technology [e.g., (10)], it can be expected that upgrading a device will be more onerous for older than younger adults. Benefits of newer versions of existing technology products, such as increased security, may also not be as salient for older users so that on balance costs of upgrading technology will outweigh benefits. Carstensen's (11) Socioemotional Selectivity Theory of life-span motivation changes also might predict that older adults would favor familiarity, staying with old devices, over exerting effort to acquire necessary information to use newer ones.

The discrepancy in ICT adoption, specifically with smartphones and computers, is noteworthy for those who posit that internet connected devices may have considerable potential for keeping older adults socially engaged (12) and possibly staving off cognitive decline (13), although the latter is a contested matter [see (14, 15)]. Mobile devices in particular are quite promising tools for health monitoring and interventions for older adults (16). Indeed, numerous electronic applications have been created specifically to aid older adults [e.g., (17)]. Mobile devices can be used to monitor health in ways that can increase effectiveness of health interventions (18). Internet connected ICT devices can also help provide health interventions that are specifically tailored to individual needs in meaningful ways (19). Unfortunately, only a limited number of older adults will be able to access these resources, given that ICT ownership among that age group is far from ubiquitous. However, lack of ownership may not be the only barrier preventing the use of these health aids. Obsolete devices, which, for the purpose of this text, are defined as those devices that are incapable of supporting a desired function that is supported by more modern devices of the same ICT class (i.e., smartphone, tablet, laptop, etc.), may present an additional barrier. Obsolete devices are also likely to be less secure and hence expose their users to hacking and to malware that can enroll their devices in Botnets (20). For instance, operating system security updates have typically become unavailable after 2 to 3 years for many Android smartphones in the USA. Very recently, Samsung decided to transition to providing 4 years of software support for most Galaxy phones and tablets (21). However, this promise will not apply retroactively to any devices released before 2019. Meanwhile, German government officials are currently negotiating with the European Union in an attempt to enforce a minimum of 7 years of support for Android and iOS devices (22). It is unclear how the negotiations will conclude, or whether devices that were manufactured earlier will be retroactively included, but it may prove difficult to enforce new laws on products that have already been released. Therefore, current trends seem to indicate meaningful extensions to device lifetimes are upcoming, but those who already own obsolete devices may still be at risk for malicious attacks. This could be particularly worrisome if a device is monitoring sensitive health information. For example, data from 2019 indicated that “two in five (40%) Android users worldwide are no longer receiving vital security updates from Google, potentially putting them at risk of data theft, ransom demands and a range of other malware attacks” (23). This alarming finding raises questions about the wisdom of housing sensitive medical information on smartphone devices for a sizable portion of the population.

While the ages of the individuals owning smartphones has been evaluated, the ages of the technological devices themselves is not well documented. At present, it is uncertain whether some demographic groups tend to have more up-to-date devices than others. For example, older adults may occasionally receive “hand-me-up” devices, passed to them by their children or relatives who have bought newer devices. If an older adult were to own a smartphone that they received second hand, or if they merely held on to their device for a long time, the kinds of services they could access might be limited and the security of the devices might be compromised. Therefore, it is important to investigate differences in the ages of technological devices owned by different demographic groups, particularly older vs. younger adults. In this study, we sampled a wide age range for ownership rather than comparing older vs. younger cohorts.

We present results from a survey of adults across the lifespan concerning the technology that they own. Survey questions asked participants about the types of technological devices they own, how long they have owned those devices, how they acquired them, and other questions about their use.

Based on the existing literature about age and technology ownership and use, we aimed to test three hypotheses:

H1: Age would be a significant negative predictor of technology ownership.

Even in a convenience sample that are users of ICT devices, we expect to replicate robust findings about technology ownership and use lagging as a function of age/birth cohort.

H2: Age would be a significant positive predictor of the age of owned technology devices.

Much as older adults are more likely to age in place in older homes that are ill-suited to their needs (24), and are more likely to own older vehicles that are not equipped with modern safety features to combat the dangers of age-related frailty (25, 26), we expect that they are less likely to refresh technology devices hence own older, less secure ones than younger adults. For the purposes of this paper, when we discuss older technology, we are referring to specific devices that are owned for a longer period of time. We are not drawing reference to the class of ICT device (desktop, laptop, smartphone etc.) and when that class of device was invented.

H3: Income would moderate the relationship between participant age and device ownership age, and the relationship between participant age and device age.

US Taxpayer average adjusted gross income tends to rise with age from the teen years to age 55–65 years, then incurs a substantial decline after the age of 65, the age when retirement from full-time work typically occurs (38). We expect that those with higher income will have fewer financial barriers for owning various technological devices and more likely to own newer devices than their lower income age-matched peers.

## Materials and methods

An online survey was administered to 407 participants across various age groups ranging from 19 to 97 (Mean = 60.6, SD = 16.9). Fifty-eight percent of participants were female, and 86% identified white as their primary racial group (For comparison, 76% of the United States population consider themselves “white alone,” (27). Median reported household income was between $60,000 and $79,999, while the median reported education level was a bachelor's degree. In the general population of the United States, median household income in 2020 was $64,994, and 33% of those 25 or older had attained a bachelor's degree or a higher level of education (27).

American participants were recruited using three separate methods, Mechanical Turk (*n* = 41), Prolific.co (*n* = 165), and a database of older adults (*n* = 201) who agreed to participate in research in association with the Institute for Successful Longevity at Florida State University. Mechanical Turk and Prolific participants were restricted to American residents using tools built into each platform which limit to whom the survey is visible. Surveys were completed using Qualtrics software.

**Figure 1:**
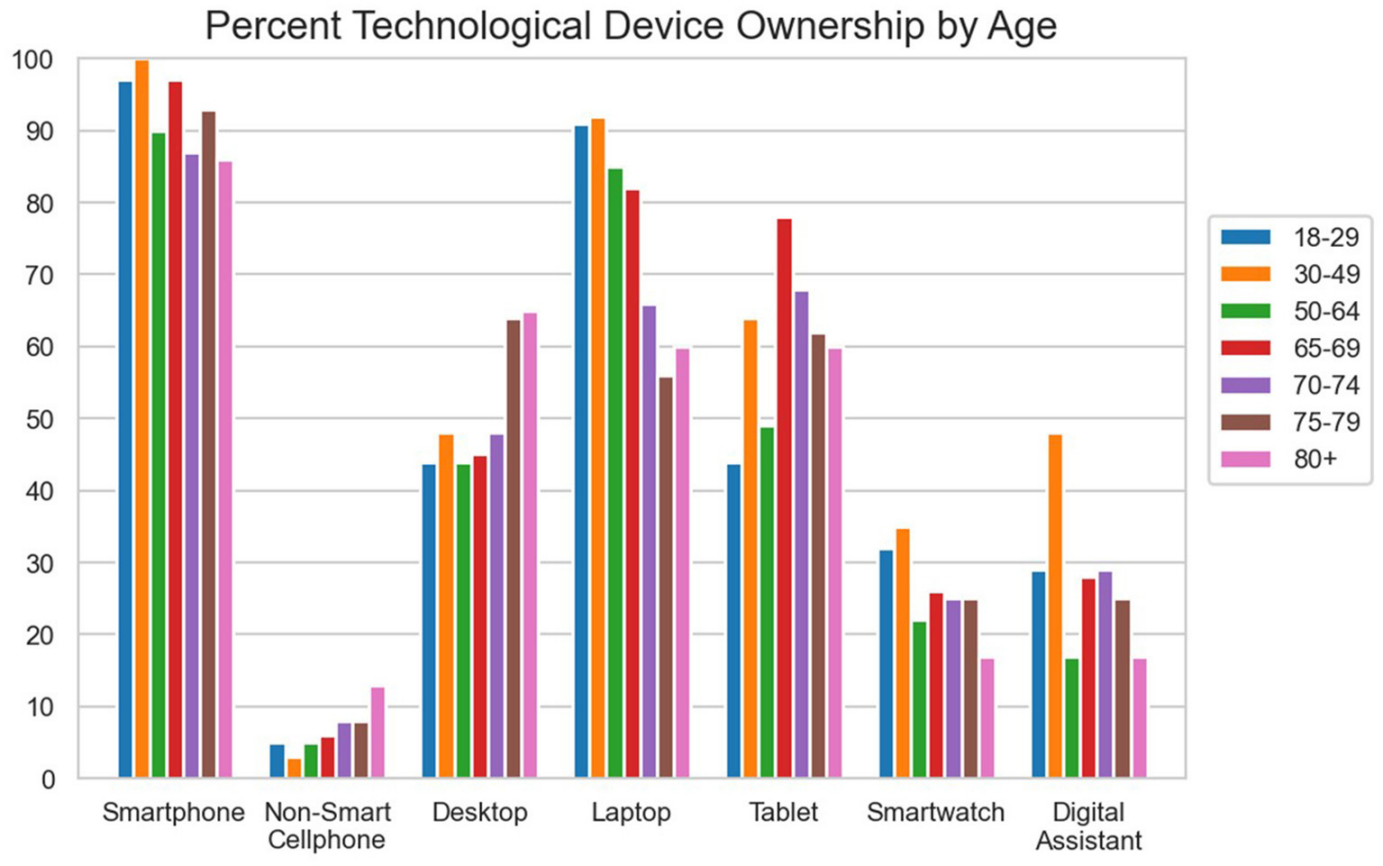
Rates of ownership of different ICT devices separated by age group.

The demographic variables of interest were age, income, gender, education, race, and marital status. Age was treated as a continuous variable in all regression models, but Figure 1 and Supplementary Table S3 report averages for age categories. Income was stratified into categories based on $10,000 increments where the first category was, “Less than $10,000,” and the final category was “$80,000 or more.” Gender was analyzed using categories “male” and “female,” as only three participants reported non-binary status. Categories of educational attainment ranged from “No formal education” to “Doctoral degree (PhD, MD, EdD, DDS, JD, etc.),” while the lowest reported level of education was “High school graduate/GED.” Due to a lack of racial diversity in this sample, race was categorized as white or non-white. Marital status was analyzed as either married or non-married, which served to indicate the likely presence of another person in the household. Note that because the data were gathered using the internet, the survey would be expected to overestimate technology ownership (technology was needed to participate), and possibly to underestimate technology obsolescence as those who own but have abandoned technology products would not have been able to participate.

Participants were asked whether they owned seven different types of technology: smartphone, mobile phone (non-smartphone), desktop computer, laptop computer, tablet, smartwatch, and home digital assistant. The age of each device owned was measured using a Likert scale in which participants selected the category that represented how long they had owned their device, from “less than 1 month,” to “8 years or more.” Participants who selected “8 years or more” for a device were asked to enter the number of years that they owned that device. A specific length of time was assigned to each categorical response, which was the midpoint of the category that was selected. For example, if a participant selected that they had owned a device for “6 months < 1 year”, a length of 9 months was assumed for that device. Demographic information can be seen in Supplementary Table S1, and correlations between demographics used as predictors in regression models can be seen in Supplementary Table S2.

Multiple logistic regression analysis was used to ascertain the relationships between the demographic variables discussed above and rates of ownership of different ICT devices. Multiple linear regression analysis was used for predicting the ages of the devices owned. Regression models were calculated in R. For each analysis, a result was deemed significant if *p* < 0.05. To create a more conservative estimate of the associations between different demographic variables and the measures of interest, any reported age of a device that was 3 or more standard deviations from the mean was not included in the analyses, helping accounting for typing errors and mistaken answers. For example, one respondent reported having owned their current smartphone for 30 years. While it may be possible that this individual owned one of the very first devices that could have been called a smartphone, created by IBM the early 1990's, it is highly unlikely that this device is still being used, especially given the fact that the frequencies that cell providers support have changed over time, many currently adopting a fifth-generation technology standard. Therefore, outliers were assessed to exclude potentially erroneous responses that could lead to overestimates of the ages of devices.

Ethics approval was attained through the Institutional Review Board (IRB) at Florida State University. This remote survey was determined to be exempt from the need to attain signed consent. Participants were presented with a description of the survey on the first screen they saw after clicking the survey link, along with contact information for the researchers and Florida State University IRB, should they have any concerns or questions.

## Results

As a test of H3 (i.e., income would moderate the relationship between a participant's age and whether they own a device or the age of that device), an interaction term for the relationship between age and income was initially added to each regression model. However, this interaction term was not significant in any model, nor did it contribute any significant improvement to model fit estimates. To ease interpretation of the main effects of the predictors in each model, the interaction term was dropped in the analyses reported here.

### Device ownership and use

Rates of ownership of each device for each age group can be seen in Figure 1 (numerical values shown in Supplementary Table S3). Logistic regression models were run to ascertain which demographic factors were associated with technology ownership (coefficient estimates and significance can be seen in Supplementary Tables S4– S10).

As predicted (H1), older age was negatively associated with ownership of smartphones, laptops, smartwatches, and home digital assistants. However, age was not associated with ownership of desktop computers or tablets.

Income was also associated with ownership of a number of devices. Higher levels of reported income had a significant positive association with ownership of smartphones and smartwatches. This association was inverted for non-smart mobile phones, where lower income was associated with higher likelihood of ownership.

Males were shown to be more likely to own desktop computers than females, yet females were more likely to own home digital assistants than males.

Some demographic measures had significant associations with certain types of devices and not others. Higher levels of education were associated with ownership of laptop computers yet were unrelated to any other device. White respondents were more likely to own home digital assistants than other races. Finally, married participants were more likely to own tablets than unmarried participants.

### Age of devices

The same demographic information used in the previous logistic regression models was used in a new set of linear regression models to predict the age of each of the technological devices currently owned by respondents. Average device ages after removal of outliers, and number of outliers removed, can be seen in Supplementary Table S11. Regression estimates can be seen in Supplementary Tables S12–S18.

As predicted, a participant's age had a significant association with the age of several of the devices that they own. Older reported chronological age was related to ownership of older smartphones, desktop computers, and laptop computers.

Female participants were more likely to have older desktop computers. Those with lower income were more likely to own older laptops. Those who were unmarried were more likely to own older smartwatches. Finally, white participants were more likely to own older smart home assistant devices (M = 2.3 years) than other races (M = 1.4 years).

Respondents were also asked whether the device that they currently use was purchased new or not (either purchased used or given to them). Age had no significant association with likelihood of getting any device new or used (*p*'s > 0.10). Also, if a participant was planning on replacing their device in the future, the participant's age was not significantly associated with how long they planned to wait until replacing their device (*p*'s > 0.05 for all types of devices). However, in a logistic regression model higher age was associated with an increased probability that a participant did not plan on replacing the smartphone (odds ratio = 1.04) or home digital assistant (odds ratio = 1.08), that they currently own.

## Discussion

Our survey results confirmed expectations (H1) that older adults would be less likely to own certain kinds of technological devices. However, this was not true for some classes of ICT devices which have been present in society for longer, such as desktop computers, tablets, or non-smart mobile phones. Desktop computers were introduced to the consumer population in the 1980's with the dawn of the microprocessor, which means that a desktop has been an integral part of society for far longer than all of the other devices in the current survey. Its sustained role in our society means that modern day older adults are likely much more familiar with their purpose and use. Tablets, on the other hand, are newer than desktop computers. While older adults have had less experience with tablets than their less portable desktop counterparts, tablets offer some usability advantages that desktop computers do not in terms of interaction *via* a direct positioning device (finger or stylus) rather than indirect positioning (e.g., mouse), though also some disadvantages (software keyboard vs. a keyboard with movable keys) (28). Tsai et al. (29) report that the touchscreen of a tablet offers a certain simplicity and ease of use for older adults, who may find a series of clicks with a mouse and inputs on a keyboard to be a bit complex if they are not familiar with the modern interface of a new desktop or laptop. Finally, the positive association between old age and ownership of a non-smart mobile phone is consistent with Pew Research data showing that although older adults are highly likely to own a cell phone, it is less likely to be a smartphone.

Critical to our hypothesis, H2, the age of our respondents was also associated with the age of a number of devices that they owned. Older respondents tended to own older smartphones, desktop computers, and laptop computers. As modern interfaces change, older adults may choose to retain the devices that they have experience with and know well given that their cost of new learning is much higher than for younger adults (10) and their expressed willingness to learn new technology is more highly discounted if they do not see value in the technology (30). Rosales and Fernández-Ardèvol (31) have also reported that older adults spend less time using the smartphones that they own, meaning that those devices may need to be replaced less often for reasons of wear and tear. However, age was not associated with whether a respondent bought any of their devices new or not, perhaps disconfirming the notion that older adults disproportionately receive older “hand-me-up” devices instead of buying them new. It may be the case that a more representative sample would show such an effect, as discussed later, but evidence for this donation process is not apparent here.

While evidence for income moderating the relationship between respondent age and device ownership or device age was not found (H3), income did have a significant positive association with ownership of several different devices. Thus, it is possible that high costs of technology could be preventing lower-income individuals from adopting certain devices. The one exception was ownership of a non-smart phone, which could be considered a less contemporary class of ICT device than the more modern smartphone. However, among those who own a given device, income was not a significant predictor of the age of the device, save for laptops. It may be the case that individuals for whom buying a certain technological device is within budget are also able to update their device on a regular basis. While some devices can be expensive, updating is an occurrence that most often happens on the scale of years, and not months, so if income is sufficient to purchase the initial device, income may not tend to be a limiting factor to updating a couple of years later. However, once again, a sample that is not collected using remote survey technology may show differing results.

The present results reveal important associations between the age of an individual and the status of the devices they own. One of the most worrisome implications of these findings could be the potential for hacking of obsolete devices that tend to be owned by older adults. Without the support of consistent software updates to protect against the most recently developed cyber threats, older adults would be more vulnerable to attacks. Older adults over age 60 reported more cases of fraud in 2020 than any other age group, and a portion of that fraud was in the form of phishing, ransomware, malware, etc., causing millions of dollars in reported losses that year (32). Even among more modern devices, protective measures are never immediate, because new threats need to be detected and countered by developers before updates can be sent out. The threats present for completely unsupported devices could be especially financially troublesome, particularly for someone on a restricted budget after retirement.

Although the noted relationships have emerged as significant, it is important to note that effect sizes, as indicated by odds ratios and regression coefficients in supplemental tables, remained modest. For example, participant age predicted smartphone age with a coefficient of 0.03, indicating that roughly 33 years of age difference between participants would result in a 1-year difference in device age. While these differences seem small in scale, they may still be meaningful. Average smartphone age for those participants aged 35 and younger was 2.15 years. One additional year in the age of a device may mean that the device is no longer supported by critical security updates. As an example, our survey data was collected in March of 2021. Nine of our respondents reported owning a pixel 3 smartphone, which received its last security update in October of 2021, when the planned 3 years of support ran out (33). Of these nine respondents, eight were over the age of 55, and six were over the age of 65. Five of the nine participants reported when they were planning on replacing their device, and only one of them reported an intent to replace it in less than a year. This means that among our respondents with this model of smartphone, there may be individuals currently unprotected by security updates.

It is also important to consider older adults' access to new applications, some of which may be specifically designed for health benefits [e.g., cognitive training or health monitoring (16)]. Obsolete and unsupported operating systems may not be capable of incorporating these newly designed applications. Even if there are workarounds for older adults to use such applications on an obsolete device, their technological proficiency is typically lower than that of younger adults (34–37), and could limit their ability to find such solutions.

While informative, this sample also has shortcomings. Our respondents are not representative of the population at large, being more highly educated and wealthier, two factors shown in national and international surveys to be positively associated with technology adoption. Thus, we likely overestimate technology ownership and underestimate the degree of technology obsolescence in the general population. We reported a much higher rate of technology ownership among older adults than is reported elsewhere, particularly for smartphones, which may be because our survey was conducted electronically. This electronic survey may have selected for participants with higher levels technology ownership and potentially more modern devices than would be seen in the general population, even among younger adults, which should be taken into consideration. The COVID-19 pandemic has limited in-person interaction with participants, particularly older adults, but conducting this survey in a manner that does not rely on an internet connected device (either in person or over the phone) should provide a more representative sample. Our participant pool also lacked racial/ethnic diversity, preventing us from making nuanced assessments of the role of race/ethnicity.

## Conclusion

This study reveals the importance of considering technology obsolescence when designing or implementing a technology-based health intervention. Older adults, many of whom can benefit from effective health monitoring and intervention technology, tend to own slightly older devices, which may put them past a critical threshold for receiving necessary security updates, particularly on mobile devices. As a result, any sensitive health information stored on these devices could be vulnerable.

While the current study indicates potential vulnerabilities, it could be an underestimation of the problem. The current sample was collected through electronic surveys, which may imply that these data represent a more technologically savvy sample than would typically exist in the larger population. For this reason, further research is needed to properly assess the broader societal impact of technology obsolescence.

## Data availability statement

The raw data supporting the conclusions of this article will be made available by the authors, without undue reservation.

## Ethics statement

The studies involving human participants were reviewed and approved by Florida State University Office For Human Subjects Protection. Written informed consent for participation was not required for this study in accordance with the national legislation and the institutional requirements.

## Author contributions

NG gained institutional ethics approval, dealt with participant recruitment, and conducted statistical analyses. Both authors collaborated on searching the literature, generating survey questions, and writing the manuscript.

## Funding

This work was supported in part by a grant from the National Institute on Aging, under the auspices of the Center for Research and Education on Aging and Technology Enhancement (CREATE), 1P01AG073090.

## Conflict of interest

The authors declare that the research was conducted in the absence of any commercial or financial relationships that could be construed as a potential conflict of interest.

## Publisher's note

All claims expressed in this article are solely those of the authors and do not necessarily represent those of their affiliated organizations, or those of the publisher, the editors and the reviewers. Any product that may be evaluated in this article, or claim that may be made by its manufacturer, is not guaranteed or endorsed by the publisher.
