# Supplementary material for: Technology obsolescence across the adult lifespan in a USA internet sample
Source: Front Public Health. 2022 Oct 6;10:1005822. doi: 10.3389/fpubh.2022.1005822 (PMC9582456; doi:10.3389/fpubh.2022.1005822)
Supplement: Supplementary file 1 [file Table_1.DOCX]

Supplementary Material

# Supplementary Tables

**Table S1**

| Participant Demographics | |
| --- | --- |
|  | Mean or Percentage (N = 407) |
| Age (19-97) | 60.6 (SD = 16.9) |
| Gender | 58% Female |
| Education | Less than HS/GED = <1% |
|  | HS/GED = 6% |
|  | Vocational Training = 3% |
|  | Some College = 22% |
|  | Bachelor = 32% |
|  | Master's = 27% |
|  | Doctoral = 9% |
| Race | White = 86% |
|  | Black = 5% |
|  | Other* = 8% |
| Household Income | Less than $10k = 1% |
|  | $10k - $19, 999 = 5% |
|  | $20k - $39, 999 = 14% |
|  | $40k - $59,999 = 20% |
|  | $60k - $79,999 = 17% |
|  | $80k + = 35% |
|  | Do not answer = 8% |
| Marital Status | Married = 54% |
| *Note.* *Asian, Native American, Alaska Native, Multi-Racial, no primary group, etc. | |

**Table S2**

| Demographic Correlations | | | | | | |
| --- | --- | --- | --- | --- | --- | --- |
|  | 1. | 2. | 3. | 4. | 5. | 6. |
| 1. Age | 1 |  |  |  |  |  |
| 2. Gender | 0.07 | 1 |  |  |  |  |
| 3. Education | 0.30^**^ | -0.07 | 1 |  |  |  |
| 4. Race | 0.34^**^ | -0.14^**^ | 0.02 | 1 |  |  |
| 5. Income | 0.15^**^ | -0.13^*^ | 0.32^**^ | 0.08 | 1 |  |
| 6. Marital Status | 0.23^**^ | -0.16^**^ | 0.13^*^ | 0.21^**^ | 0.37^**^ | 1 |

*Note.* Gender: 0 = male 1= female. Race: 0 = non-white, 1 = white. Marital Status: 0 = non-married, 1 = married. ^*^ p < .05, ^**^ p < .01 (2-tailed).

**Table S3**

|  | Technological Device Ownership Rates | | | | | | |
| --- | --- | --- | --- | --- | --- | --- | --- |
|  | Device | | | | | | |
| Age Group | Smartphone | Cellphone (Non-smartphone) | Desktop Computer | Laptop Computer | Tablet | Smartwatch | Home Digital Assistant |
| 18-29 (n=34) | 97% | 5% | 44% | 91% | 44% | 32% | 29% |
| 30-49 (n=56) | 100% | 3% | 48% | 92% | 64% | 35% | 48% |
| 50-64 (n=95) | 90% | 5% | 44% | 85% | 49% | 22% | 17% |
| 65-69 (n=75) | 97% | 6% | 45% | 82% | 78% | 26% | 28% |
| 70-74 (n=74) | 87% | 8% | 48% | 66% | 68% | 25% | 29% |
| 75-79 (n=48) | 93% | 8% | 64% | 56% | 62% | 25% | 25% |
| 80+ (n=23) | 86% | 13% | 65% | 60% | 60% | 17% | 17% |
| Total (N=405) | 93% | 6% | 49% | 78% | 62% | 26% | 27% |

*Note.* Two of the 407 participants did not report age, leaving N=405 for all age-related analyses

| \| **Table S4** \| Smartphone Ownership \| \| \| \| \|  \| \| --- \| --- \| --- \| --- \| --- \| --- \| --- \| \| Predictor \| B \| Std. Error B \| OR \| Z \| Sig. \| \| \| Age \| -0.06 \| 0.02 \| 0.94 \| -2.89 \| 0.00^**^ \| \| \| Income \| 0.54 \| 0.20 \| 1.72 \| 2.76 \| 0.01^**^ \| \| \| Gender \| -0.01 \| 0.49 \| 0.99 \| -0.03 \| 0.98 \| \| \| Education \| 0.09 \| 0.19 \| 1.09 \| 0.47 \| 0.64 \| \| \| Race \| 0.67 \| 0.71 \| 1.96 \| 0.95 \| 0.34 \| \| \| Marital Status \| -0.19 \| 0.50 \| 0.83 \| -0.38 \| 0.70 \| \| | \| **Table S5** \| Non-Smart Mobile Phone Ownership \| \| \| \| \|  \| \| --- \| --- \| --- \| --- \| --- \| --- \| --- \| \| Predictor \| B \| Std. Error B \| OR \| Z \| Sig. \| \| \| Age \| 0.03 \| 0.02 \| 1.03 \| 1.77 \| 0.08**_._** \| \| \| Income \| -0.57 \| 0.19 \| 0.57 \| -2.98 \| 0.00^**^ \| \| \| Gender \| -0.50 \| 0.47 \| 0.60 \| -1.07 \| 0.29 \| \| \| Education \| 0.02 \| 0.19 \| 1.02 \| 0.12 \| 0.90 \| \| \| Race \| -0.41 \| 0.70 \| 0.67 \| -0.58 \| 0.56 \| \| \| Marital Status \| -0.44 \| 0.51 \| 0.65 \| -0.86 \| 0.39 \| \| |
| --- | --- | --- | --- | --- | --- | --- | --- | --- | --- | --- | --- | --- | --- | --- | --- | --- | --- | --- | --- | --- | --- | --- | --- | --- | --- | --- | --- | --- | --- | --- | --- | --- | --- | --- | --- | --- | --- | --- | --- | --- | --- | --- | --- | --- | --- | --- | --- | --- | --- | --- | --- | --- | --- | --- | --- | --- | --- | --- | --- | --- | --- | --- | --- | --- | --- | --- | --- | --- | --- | --- | --- | --- | --- | --- | --- | --- | --- | --- | --- | --- | --- | --- | --- | --- | --- | --- | --- | --- | --- | --- | --- | --- | --- | --- | --- | --- | --- | --- | --- | --- | --- | --- | --- | --- | --- | --- | --- | --- | --- | --- | --- | --- | --- |
| \| **Table S6** \| Desktop Computer Ownership \| \| \| \| \|  \| \| --- \| --- \| --- \| --- \| --- \| --- \| --- \| \| Predictor \| B \| Std. Error B \| OR \| Z \| Sig. \| \| \| Age \| 0.01 \| 0.01 \| 1.01 \| 1.08 \| 0.28 \| \| \| Income \| 0.10 \| 0.09 \| 1.11 \| 1.09 \| 0.28 \| \| \| Gender \| -0.45 \| 0.22 \| 0.64 \| -2.01 \| 0.04^*^ \| \| \| Education \| -0.09 \| 0.09 \| 0.91 \| -1.00 \| 0.32 \| \| \| Race \| 0.38 \| 0.37 \| 1.47 \| 1.04 \| 0.30 \| \| \| Marital Status \| 0.02 \| 0.24 \| 1.02 \| 0.09 \| 0.93 \| \| | \| **Table S7** \| Laptop Computer Ownership \| \| \| \| \|  \| \| --- \| --- \| --- \| --- \| --- \| --- \| --- \| \| Predictor \| B \| Std. Error B \| OR \| Z \| Sig. \| \| \| Age \| -0.06 \| 0.01 \| 0.94 \| -4.95 \| 0.00^**^ \| \| \| Income \| -0.13 \| 0.13 \| 0.87 \| -1.07 \| 0.28 \| \| \| Gender \| 0.28 \| 0.28 \| 1.33 \| 1.01 \| 0.31 \| \| \| Education \| 0.33 \| 0.12 \| 1.39 \| 2.82 \| 0.00^**^ \| \| \| Race \| 0.34 \| 0.52 \| 1.40 \| 0.65 \| 0.52 \| \| \| Marital Status \| -0.11 \| 0.30 \| 0.90 \| -0.35 \| 0.72 \| \| |
| \| **Table S8** \| Tablet Ownership \| \| \| \| \|  \| \| --- \| --- \| --- \| --- \| --- \| --- \| --- \| \| Predictor \| B \| Std. Error B \| OR \| Z \| Sig. \| \| \| Age \| 0.01 \| 0.01 \| 1.01 \| 1.30 \| 0.19 \| \| \| Income \| 0.16 \| 0.09 \| 1.17 \| 1.65 \| 0.10**_._** \| \| \| Gender \| 0.44 \| 0.23 \| 1.55 \| 1.87 \| 0.06**_._** \| \| \| Education \| -0.02 \| 0.10 \| 0.98 \| -0.16 \| 0.87 \| \| \| Race \| 0.32 \| 0.36 \| 1.37 \| 0.87 \| 0.39 \| \| \| Marital Status \| 0.52 \| 0.25 \| 1.68 \| 2.10 \| 0.04^*^ \| \| | \| **Table S9** \| Smartwatch Ownership \| \| \| \| \|  \| \| --- \| --- \| --- \| --- \| --- \| --- \| --- \| \| Predictor \| B \| Std. Error B \| OR \| Z \| Sig. \| \| \| Age \| -0.02 \| 0.01 \| 0.98 \| -2.32 \| 0.02^*^ \| \| \| Income \| 0.27 \| 0.11 \| 1.31 \| 2.46 \| 0.01^*^ \| \| \| Gender \| 0.44 \| 0.26 \| 1.56 \| 1.73 \| 0.08**_._** \| \| \| Education \| 0.17 \| 0.11 \| 1.18 \| 1.54 \| 0.12 \| \| \| Race \| -0.08 \| 0.40 \| 0.92 \| -0.20 \| 0.84 \| \| \| Marital Status \| 0.27 \| 0.27 \| 1.31 \| 0.99 \| 0.32 \| \| |

| **Table S10** | Home Digital Assistant Ownership | | | | |  |
| --- | --- | --- | --- | --- | --- | --- |
| Predictor | B | Std. Error B | OR | Z | Sig. | |
| Age | -0.03 | 0.01 | 0.97 | -3.55 | 0.00^**^ | |
| Income | 0.20 | 0.11 | 1.22 | 1.91 | 0.06**_._** | |
| Gender | 0.51 | 0.25 | 1.67 | 2.02 | 0.04^*^ | |
| Education | 0.12 | 0.11 | 1.13 | 1.14 | 0.25 | |
| Race | 1.08 | 0.47 | 2.94 | 2.32 | 0.02^*^ | |
| Marital Status | 0.47 | 0.27 | 1.59 | 1.70 | 0.09**_._** | |

*Note.* Gender: 0 = male 1= female. Race: 0 = non-white, 1 = white. Marital Status: 0 = non-married, 1 = married. ^**^ < .001 , ^*^ < .05 , **_._** < .10 , OR = Odds Ratio

**Table S11**

|  | Age of Device (Years) | | |  |
| --- | --- | --- | --- | --- |
|  | Mean | | SD | # Outliers Removed |
| Smartphone | 2.84 | 2.33 | | 5 |
| Cellphone (Non-smartphone) | 6.70 | 5.49 | | 1 |
| Desktop Computer | 5.05 | 4.11 | | 6 |
| Laptop Computer | 3.93 | 2.89 | | 5 |
| Tablet | 3.76 | 2.33 | | 3 |
| Smartwatch | 1.86 | 1.39 | | 1 |
| Home Digital Assistant | 2.21 | 1.32 | | 4 |

*Note.* Mean and standard deviation values for the age of each device are determined after outliers are removed. Device ages are averaged across all demographic categories.

| \| **Table S12** \| Smartphone Age \| \| \| \| \|  \| \| --- \| --- \| --- \| --- \| --- \| --- \| --- \| \| Predictor \| B \| Std. Error B \| Β \| Z \| Sig. \| \| \| Age \| 0.03 \| 0.01 \| 0.23 \| 3.72 \| 0.00^**^ \| \| \| Income \| -0.02 \| 0.11 \| -0.01 \| -0.15 \| 0.88 \| \| \| Gender \| 0.16 \| 0.26 \| 0.03 \| 0.61 \| 0.54 \| \| \| Education \| 0.14 \| 0.11 \| 0.07 \| 1.26 \| 0.21 \| \| \| Race \| -0.20 \| 0.42 \| -0.03 \| -0.47 \| 0.64 \| \| \| Marital Status \| -0.16 \| 0.28 \| -0.03 \| -0.55 \| 0.58 \| \| | \| **Table S13** \| Non-Smart Mobile Phone Age \| \| \| \| \|  \| \| --- \| --- \| --- \| --- \| --- \| --- \| --- \| \| Predictor \| B \| Std. Error B \| β \| Z \| Sig. \| \| \| Age \| 0.18 \| 0.09 \| 0.47 \| 1.88 \| 0.09**_._** \| \| \| Income \| 0.23 \| 1.11 \| 0.06 \| 0.21 \| 0.84 \| \| \| Gender \| 2.30 \| 3.00 \| 0.21 \| 0.77 \| 0.46 \| \| \| Education \| -0.42 \| 1.08 \| -0.11 \| -0.39 \| 0.71 \| \| \| Race \| 2.94 \| 5.01 \| 0.17 \| 0.59 \| 0.57 \| \| \| Marital Status \| 1.98 \| 3.08 \| 0.18 \| 0.64 \| 0.53 \| \| |
| --- | --- | --- | --- | --- | --- | --- | --- | --- | --- | --- | --- | --- | --- | --- | --- | --- | --- | --- | --- | --- | --- | --- | --- | --- | --- | --- | --- | --- | --- | --- | --- | --- | --- | --- | --- | --- | --- | --- | --- | --- | --- | --- | --- | --- | --- | --- | --- | --- | --- | --- | --- | --- | --- | --- | --- | --- | --- | --- | --- | --- | --- | --- | --- | --- | --- | --- | --- | --- | --- | --- | --- | --- | --- | --- | --- | --- | --- | --- | --- | --- | --- | --- | --- | --- | --- | --- | --- | --- | --- | --- | --- | --- | --- | --- | --- | --- | --- | --- | --- | --- | --- | --- | --- | --- | --- | --- | --- | --- | --- | --- | --- | --- | --- |
| \| **Table S14** \| Desktop Computer Age \| \| \| \| \|  \| \| --- \| --- \| --- \| --- \| --- \| --- \| --- \| \| Predictor \| B \| Std. Error B \| Β \| Z \| Sig. \| \| \| Age \| 0.07 \| 0.02 \| 0.33 \| 4.09 \| 0.00^**^ \| \| \| Income \| 0.22 \| 0.25 \| 0.07 \| 0.90 \| 0.37 \| \| \| Gender \| 1.31 \| 0.57 \| 0.17 \| 2.31 \| 0.02^*^ \| \| \| Education \| -0.03 \| 0.24 \| -0.01 \| -0.12 \| 0.91 \| \| \| Race \| -1.58 \| 1.01 \| -0.12 \| -1.57 \| 0.12 \| \| \| Marital Status \| -0.64 \| 0.62 \| -0.08 \| -1.02 \| 0.31 \| \| | \| **Table S15** \| Laptop Computer Age \| \| \| \| \|  \| \| --- \| --- \| --- \| --- \| --- \| --- \| --- \| \| Predictor \| B \| Std. Error B \| β \| Z \| Sig. \| \| \| Age \| 0.03 \| 0.01 \| 0.17 \| 2.50 \| 0.01^*^ \| \| \| Income \| -0.28 \| 0.14 \| -0.13 \| -1.98 \| 0.05^*^ \| \| \| Gender \| 0.35 \| 0.36 \| 0.06 \| 0.96 \| 0.34 \| \| \| Education \| 0.22 \| 0.15 \| 0.09 \| 1.47 \| 0.14 \| \| \| Race \| 0.41 \| 0.56 \| 0.05 \| 0.73 \| 0.47 \| \| \| Marital Status \| -0.40 \| 0.38 \| -0.07 \| -1.05 \| 0.30 \| \| |
| \| **Table S16** \| Tablet Age \| \| \| \| \|  \| \| --- \| --- \| --- \| --- \| --- \| --- \| --- \| \| Predictor \| B \| Std. Error B \| Β \| Z \| Sig. \| \| \| Age \| 0.02 \| 0.01 \| 0.12 \| 1.64 \| 0.10 \| \| \| Income \| 0.14 \| 0.15 \| 0.08 \| 0.97 \| 0.34 \| \| \| Gender \| 0.38 \| 0.33 \| 0.08 \| 1.14 \| 0.25 \| \| \| Education \| -0.02 \| 0.14 \| -0.01 \| -0.13 \| 0.90 \| \| \| Race \| -0.41 \| 0.58 \| -0.05 \| -0.71 \| 0.48 \| \| \| Marital Status \| -0.32 \| 0.37 \| -0.07 \| -0.86 \| 0.39 \| \| | \| **Table S17** \| Smartwatch Age \| \| \| \| \|  \| \| --- \| --- \| --- \| --- \| --- \| --- \| --- \| \| Predictor \| B \| Std. Error B \| β \| Z \| Sig. \| \| \| Age \| 0.01 \| 0.01 \| 0.07 \| 0.61 \| 0.55 \| \| \| Income \| -0.03 \| 0.13 \| -0.03 \| -0.23 \| 0.82 \| \| \| Gender \| 0.22 \| 0.29 \| 0.08 \| 0.75 \| 0.46 \| \| \| Education \| 0.23 \| 0.12 \| 0.20 \| 1.87 \| 0.06**_._** \| \| \| Race \| 0.27 \| 0.44 \| 0.07 \| 0.63 \| 0.53 \| \| \| Marital Status \| -0.82 \| 0.31 \| -0.30 \| -2.61 \| 0.01^*^ \| \| |

| **Table S18** | Home Digital Assistant Age | | | | |  |
| --- | --- | --- | --- | --- | --- | --- |
| Predictor | B | Std. Error B | β | Z | Sig. | |
| Age | 0.01 | 0.01 | 0.10 | 0.84 | 0.41 | |
| Income | 0.04 | 0.13 | 0.03 | 0.31 | 0.76 | |
| Gender | 0.44 | 0.29 | 0.16 | 1.53 | 0.13 | |
| Education | 0.00 | 0.12 | 0.00 | 0.03 | 0.98 | |
| Race | 1.10 | 0.54 | 0.22 | 2.04 | 0.04^*^ | |
| Marital Status | -0.35 | 0.30 | -0.13 | -1.19 | 0.24 | |

*Note.* Gender: 0 = male 1= female. Race: 0 = non-white, 1 = white. Marital Status: 0 = non-married, 1 = married. ^**^ < .001 , ^*^ < .05 , **_._** < .10
